# Polysaccharide-Based Edible Coatings Containing Cellulase for Improved Preservation of Meat Quality during Storage

**DOI:** 10.3390/molecules22030390

**Published:** 2017-03-02

**Authors:** Anna Zimoch-Korzycka, Andrzej Jarmoluk

**Affiliations:** Department of Animal Products Technology and Quality Management, Wrocław University of Environmental and Life Sciences, 37 Chelmonskiego St., 51-630 Wrocław, Poland; anna.zimoch-korzycka@upwr.edu.pl

**Keywords:** chitosan, hydroxypropyl methylcellulose, cellulase, meat quality, microbiology

## Abstract

The objectives of this study were to optimize the composition of edible food coatings and to extend the shelf-life of pork meat. Initially, nine meat samples were coated with solutions containing chitosan and hydroxypropyl methylcellulose at various cellulase concentrations: 0%, 0.05%, and 0.1%, stored for 0, 7, and 14 days. Uncoated meat served as the controls. The samples were tested for pH, water activity (a_w_), total number of microorganisms (TNM), psychrotrophs (P), number of yeast and molds (NYM), colour, and thiobarbituric acid-reactive substances (TBARS). The pH and a_w_ values varied from 5.42 to 5.54 and 0.919 to 0.926, respectively. The reductions in the TNM, P, and NYM after 14 days of storage were approximately 2.71 log cycles, 1.46 log cycles, and 0.78 log cycles, respectively. The enzyme addition improved the stability of the red colour. Significant reduction in TBARS was noted with the inclusion of cellulase in the coating material. Overall, this study provides a promising alternative method for the preservation of pork meat in industry.

## 1. Introduction

Raw meat is highly perishable because it is a good source of nutrients for microorganisms. Microbial growth is the major factor that causes meat spoilage. Unfavourable changes may also occur due to chemical reactions such as lipid and myoglobin oxidation, leading to odour changes and brown colouration of meat, respectively. Biochemical reactions also may lead to moisture loss during storage. These changes are reflected in the texture or flavour of the meat [1].

Edible coatings may improve the quality and extend the shelf-life of fresh meat and meat products. Three types of coatings have been used for this purpose. These coatings are the lipid, protein, and polysaccharide coatings. Two of the most abundant polysaccharides in nature, cellulose and chitosan, are used in the production of films and coatings. Cellulose is composed of glucose with β-1,4 glycosidic linkages that are very chemically stable, but the native form is insoluble in water. Ethers of cellulose are soluble and are produced by the partial substitution of the hydroxyl groups at positions 2, 3, and 6 of the glucosyl units. The hydroxypropyl methylcellulose (HPMC) is water-soluble and shows good film-forming ability [1]. Chitosan (CH), a deacetylated chitin consisting of β-(1,4)-2-amino-d-glucopyranose and of β-(1,4)-2-acetamido-d-glucopyranose [2], with or without other additives, has been shown to have profitable applications in meat preservation [3,4,5]. Cellulase is a cellulose-degrading lytic enzyme. The presence of similar bonds in the chains of HPMC and CH ensures their enzymatic digestion by cellulase [6]. Chitooligomers produced during chemical or enzymatic hydrolysis of chitosan display more potent bactericidal effects than chitosan [5,7]. When compared to its effect on Gram-positive bacteria, the lower molecular weight chitosans exert a more potent bactericidal effect on Gram-negative bacteria [8]. The antimicrobial activity of the chitooligomers is related to the adsorption of the polymers onto the bacterial cell surface, increasing the permeability of the cell membrane and causing cell death through the loss of essential cellular materials [9].

To the best of our knowledge, the usage of a hydrolytic enzyme (except lysozyme) as an additive in polysaccharide meat coatings for improving its antimicrobial activity is novel. Our FT-IR analysis confirmed the hydrolysis of chitosan by cellulase and showed that the physicochemical properties of such films were comparable to that reported by a previous study [10]. The objective of this study was to optimize the composition of edible food coatings based on hydroxypropyl methylcellulose, chitosan, and cellulase, and to improve the shelf-life and quality of pork meat. Meat quality was assessed from the total number of microorganisms (TNM), number of psychrotrophs (P), number of yeast and mold (NYM), as well as by measuring the colour and thiobarbituric acid reactive substances (TBARS).

## 2. Results and Discussion

### 2.1. Microbiology

**Figure 1:**
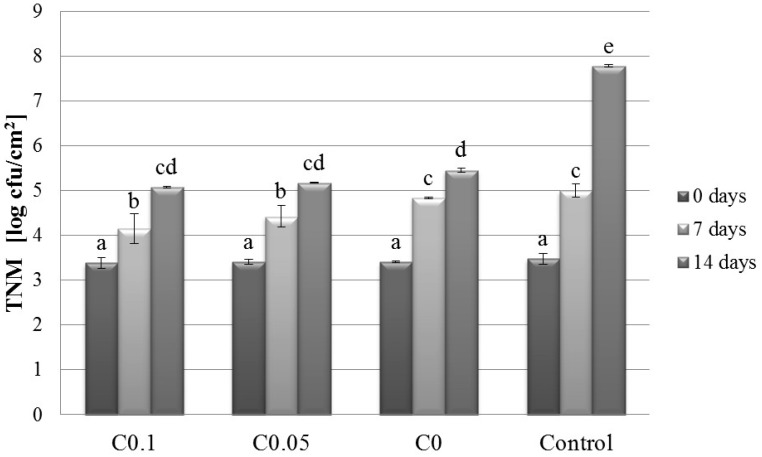
Effect of cellulase concentration and storage time on the total number of microorganisms in pork meat. Results are expressed as the mean ± standard error. Values with different letters (a–e) within the same column differ significantly (*p* < 0.05). Control–uncoated meat; C0–0% cellulase; C0.05–0.05% cellulase; C0.1–0.1% cellulase.

**Figure 2:**
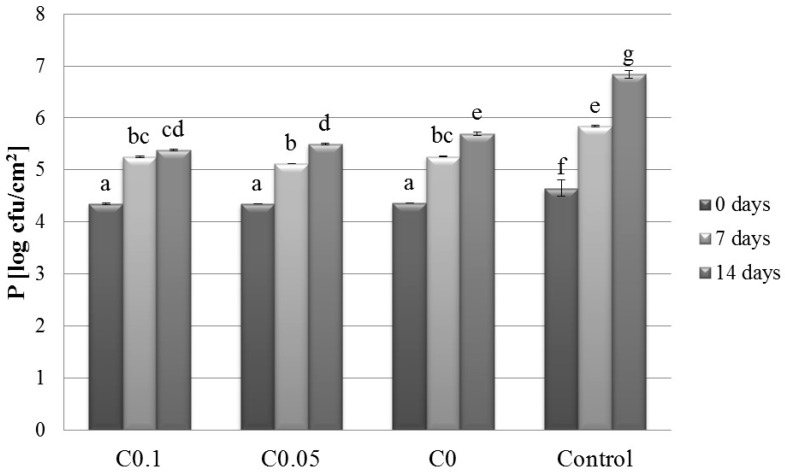
Effect of cellulase concentration and storage time on psychrotrophs population of meat pork. Results are expressed as the mean ± standard error. Values with different letters (a–g) within the same column differ significantly (*p* < 0.05). Control–uncoated meat; C0–0% cellulase; C0.05–0.05% cellulase; C0.1–0.1% cellulase.

**Figure 3:**
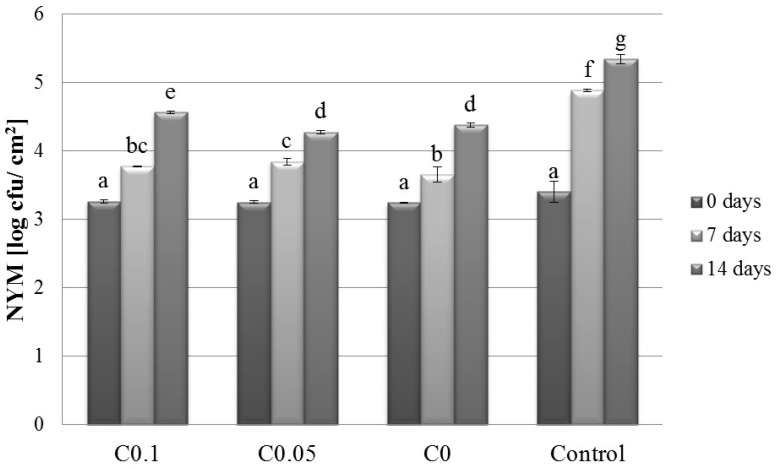
Effect of cellulase concentration and storage time on the number of yeast and mold in pork meat. Results are expressed as the mean ± standard error. Values with different letters (a–g) within the same column differ significantly (*p* < 0.05). Control–uncoated meat; C0–0% cellulase; C0.05–0.05% cellulase; C0.1–0.1% cellulase.

The effects of cellulase addition and storage time on the TNM, P, and NYM values are presented in Figure 1, Figure 2 and Figure 3, respectively. The results showed the simultaneous influence of cellulase and storage time on TNM, P, and NYM. The TNM, P, and NYM were 3.48 log CFU/cm^2^, 4.65 log CFU/cm^2^, and 3.40 log CFU/cm^2^, respectively, at time 0 of storage. After 7 days of storage, these values increased to 5.00 log CFU/cm^2^, 5.84 log CFU/cm^2^, and 4.88 log CFU/cm^2^ for TNM, P, and NYM, respectively. The final TNM, P, and NYM values were 7.78 log CFU/cm^2^, 6.84 log CFU/cm^2^, and 5.34 log CFU/cm^2^, respectively. Malinowska-Pańczyk and Kołodziejska have reported total bacterial counts of 4.3 log CFU/g, 5.8 log CFU/g, and 7.4 log CFU/g, and psychrotroph counts of 4.0 log CFU/g, 5.3 log CFU/g, and 8.2 log CFU/g in minced pork after 0, 2, and 6 days of refrigerated storage, respectively [11]. Although these authors applied 60 MPa pressure at −5 °C to extend the shelf-life, the change in the bacterial count after 2 and 6 days of storage was insignificant compared to the untreated sample. The application of 193 MPa pressure (at −20 °C) caused a drop in the total bacterial count to 4.1 log CFU/g and 5.9 log CFU/g and in the psychrotroph count to 2.6 log CFU/g and 4.9 log CFU/g after 2 and 6 days, respectively. Bacterial growth in pieces of meat can be more readily controlled compared with that in minced meat. At time 0 of storage, the cellulase dose had no effect on the amounts of the tested groups of microorganisms in the samples. However, compared with the controls, the TNM dropped by 0.85 log cycles and 2.71 log cycles at 0.1% cellulase concentration after 7 and 14 days of storage, respectively. Zhao et al. analysed the microbial growth in vacuum-packed pork during its cold storage and noted a similar reduction (2.27 CFU/g) after 14 days [12]. The inhibitory effect is related to the presence of chitosan in the coating material. Chitosan inhibits the growth of bacteria, fungi, and yeast [13]. It exerts a particularly potent inhibitory effect against bacteria [14]. Cellulase has been shown to hydrolyse chitosan resulting in the production of chitooligomers [10]. Available evidence suggests that chitooligomers may have better antimicrobial properties than chitosan [15].

The psychrotrophic bacteria in the meat were sensitive to the simultaneous effects of cellulase addition in separated periods of time, and the value of *p* was 0.59 log cycles lower in the C0.1T7 sample than that determined for the Control T7 sample. A similar decrease was also noted after 14 days (1.46 log CFU/g). Psychrotrophic bacteria can grow at temperatures below 7 °C. Caldara et al. observed that these microorganisms grew faster in meat during the season when the atmospheric temperature was high [16]. Compared with the controls, the NYM at 0.1% cellulase concentration reflected a reduction of approximately 1.11 log cycles and 0.76 log cycles after 7 and 14 days of storage, respectively. Some yeasts tolerate acidic pH below 5.5 and are able to grow at temperatures above 0 °C. Molds are also able to grow at temperatures slightly above freezing [17]. On the other hand, Caldara et al., who investigated normal meat and PSE meat for yeast and mold growth, found no significant differences during the storage period [16]. The authors reported that the number of these microorganisms increased by 4.73 log cycles in the normal meat and by 5.26 log cycles in the PSE meat after 10 days of storage, with the numbers falling after this time.

The inhibitory effect of added cellulase is because of its ability to hydrolyse chitosan. Chantarasapatorn et al. found that oligochitosans act as effective food preservatives in a minced pork model [18]. These authors noted that the water soluble oligochitosans significantly improved antimicrobial activity and extended the shelf-life while maintaining the meat quality. Additionally, chitosan can be dissolved in simple acid solution, and it appears that the presence of lactic acid in the coating material can potentially have a beneficial effect. The simultaneous effect of all of these factors may have contributed to the microbial growth reduction. This fits the definition of the hurdles technology, which is commonly used for preserving food products. This technology exploits the synergistic action of many factors, resulting in robust microbial growth inhibition, whereas each factor acting alone would be less effective [19]. The sensory changes are noticeable at 10^6^ to 10^9^ CFU of spoilage microorganisms/g of meat [20]. On the other hand, the bacterial counts are still at an acceptable level when their amount is less than 10^6^ CFU/g [12]. Based on these limits, it is clear that the shelf-life of fresh pork meat was extended and the chitooligomers effectively inhibited the microbial growth.

### 2.2. Physico-Chemical Parameters

The results on the interaction effects of the physico-chemical parameters (pH, a_w_, L*, a*, b*, TBARS) are presented in Table 1. Cellulase addition and storage time significantly affected the pH value. Fourteen days of storage at 0.1% cellulase resulted in increased pH compared with the sample C0T0. Zhao et al. have reported that the pH of the vacuum-packed pork during cold storage increased from 5.72 to 5.99 after 0 and 21 days, respectively [12]. The initial pH of the Control (meat) T0 was 5.52, and this value remained nearly unchanged during the storage. It appeared that the pH was stabilized by other components of the coating material, such as chitosan or lactic acid. During the storage, the pH changed to a higher value due to the buffering capacity of meat. The pH is an important factor in meat quality assessment. The normal muscle pH is in the range of 5.5–5.7. The acidic environment of the meat does not support the growth of microorganisms except lactic acid bacteria, which are able to grow under such conditions and cause spoilage. The genera *Lactobacillus*, *Leuconostoc*, and *Carnobacterium* are dominant under these conditions [17]. The lack of air at this range of pH restricts the growth of *Pseudomonas*, *Enetrobacteriacea*, *Brochothrix tchermosphacta*, *Aeromonas*, and *Alteromonas putrefaciens* at 0–5 °C [21]. On the other hand, Garcia-Lopez et al. reported the growth of *Shewanella putrefaciens*, *Alcaligenes* spp., *Aeromonas* spp., and other species of *Enterobacteriaceae* at pH below 6 [22].

The a_w_ values are presented in Table 1. The water activity of the coated pork was mainly affected by storage time and slightly affected by the enzyme addition. The range of a_w_ in the interactions was 0.921–0.926. The growth of microorganisms is possible only in the presence of water. Most pathogenic bacteria grow faster when the water activity is between 0.90–0.99 [23]. The water activity of normal fresh pork meat is in the range of 0.98–0.99. Leistner reported that meat and meat products easily perish at a_w_ ˃ 0.95 and pH ˃ 5.2 [24]. In comparison with bacteria and yeast, which grow at an a_w_ of 0.88, fungi can grow at lower a_w_ values of 0.80 and 0.91.

Results from the analysis of interactions confirmed the simultaneous effect of cellulase and storage time on L*, a*, and b*. Samples became lighter with the prolonged storage time and darker with the rising concentration of cellulase. The colour changes were likely related to the reduction in oxygen tension, caused by vacuum-packing. Grajales-Lagunes et al. have reported that lactic acid does not contribute to the colour changes in pork [25]. The lightness of the initial meat sample was 58.62 and after 14 days of storage was 38.01. The C0T0 sample showed an L value of 53.91 and reached 59.63 in the final sample R10T14. This could be related to the creation of a transparent and bright film by HPMC and CH. In general, higher L* values are desirable. The decreased lightness value of the meat during storage is generally associated with an unattractive brown colour. This colour is due to the oxidation of red oxymyoglobin to metmyoglobin [26]. We also observed a reduction in the redness from 8.57 of the Control (meat) T0 to 6.89 of C0T0 and 6.30 of C0.1T14. However, the Control (meat) T0, C0T0, C0T0, and C0.1T14 were still in the common homogenous group, confirming the stability of redness. The a* value of the meat sample coated with material containing cellulase was significantly higher compared to the Control (meat) T7 and Control (meat) T14. Jo et al. (2001) reported that chitosan oligomer addition increased the Hunter colour a*-value of pork sausage after 1 and 2 weeks of storage with aerobic packaging [27]. The antioxidant potential of chitosan could improve the redness stability of pork slices. Zahran reported that when compared with meat dipped in water and stored for the same duration, chitosan oligomers produced by 100 kGy irradiation caused significant improvement in the a*-value of meat slices during 14 days of storage [28]. Mokhtar et al. reported that both chitosan and chitosan in combination with rosemary extract improved the red colour of fresh beef patties [29]. Beef samples became more yellow with storage and cellulase addition. The colour of meat and meat products is an important factor influencing the consumer’s purchase decision. Therefore, the deterioration of the colour of meat and meat products is a serious problem in the meat industry.

The effect of both factors on TBARS values is clearly observed. Cellulase addition and storage time significantly lowered the TBARS values. The initial TBARS values of the meat was 0.26 mg MDA/kg, which increased to 0.97 and 1.54 mg MDA/kg after 7 and 14 days of storage, respectively. The TBARS of all coated samples at time 0 was approximately 0.25–0.26 mg MDA/kg. After 7 days of storage, these values significantly increased to 0.53–0.68 mg MDA/kg, reaching 1.05–1.28 mg MDA/kg after 14 days of storage. An increase in the concentration of cellulase resulted in significantly reduced TBARS values after 7 and 14 days of storage. This reduction in TBARS was also observed in the case of meat coated without cellulase, but the magnitude of the change was smaller. Zahran reported that there was no significant differences between the MDA values of beef slices dipped in chitosan and chitosan oligomers [28]. Although Chantarasataporn et al. found a significant reduction in TBARS of minced meat treated with oligochitosans, their concentration had no effect on the TBARS value [18]. Free radicals that occur in lipid oxidation are secondary products and may be investigated by the TBARS method. The amino group of deacetylated chitosan may interact with free radicals and form stable macromolecules, inhibiting lipid oxidation [30].

## 3. Materials and Methods

### 3.1. Materials

Low-molecular-weight chitosan (DD = 75%–85%), dl-lactic acid (85% syrup) and 99% glycerol were obtained from Sigma Aldrich, Poznań, Poland. Hydroxypropyl methylcellulose, Methocel™, was purchased from Dow Chemical Co., Midland, MI, USA. Cellulase CP CONC (C) with an activity of 120 U/mg and side activity (typical) of 30 U/mg of β***-***glucanase was produced by the fermentation of non-GMO *Trichoderma longibrachiatum* (formerly *Trichoderma reesei*) that was obtained from Dyadic (Jupiter, FL, USA).

### 3.2. Coat-Forming Solution

The 2% HPMC stock solution was dissolved in distilled water and the CH stock solution was solubilized in 1% (*v*/*v*) aqueous lactic acid (LA) to the concentration of 4%. Cellulase stock solution (C) was prepared by dissolving the material in distilled water, followed by centrifugation (5000 *g*). Glycerol (G) was added such that its amount was 25% *w*/*w* of the dry weight of the polymers used. The HPMC (50 mL) and CH (25 mL) stock solutions were blended with G and C (at three different concentrations: 0%, 0.05% and 0.1%; *w*/*w*) and distilled water was added to increase the volume to 100 mL. The final concentrations of CH, HPMC, and LA in the coatings were 1%, 1%, and 0.25%, respectively (Table 2). The pH values of the coat-forming solutions C0T0–C0.1T14 were determined using a pH-meter (Handylab Schott Instruments, Mainz, Germany) and was 5.45 ± 0.08.

### 3.3. Meat Sampling

Three pig *longissimus thoracis* (LT) muscle samples were obtained within 48 h of slaughtering from a commercial slaughterhouse and sliced into twelve 20 ± 0.3-mm thick portions. The meat slices were sprayed with the desired coat-forming solutions, vacuum packed in PA/PE (polyamide/polyethylene) foil pouches, and stored at 4 °C in a cold room. The samples were analysed after 0, 7, and 14 days (Table 2) for pH, a_w_, TNM, P, NYM, colour, and TBARS. Each sample was tested for all parameters. The pH values were determined using a pH-meter (Handylab Schott Instruments) equipped with a flat-tip pH electrode for surfaces (Hanna Instruments, Woonsocket, RI, USA). The water activity was determined using an AW LAB Set H a_w_-meter (Novasina AG). Samples of meat slices, cylindrical in shape with a diameter of 38 mm and a height of 10 mm, were cut and transferred into the disposable cup of the water activity meter such that the vapour from the coated meat was in equilibrium with the surrounding air.

### 3.4. Microbial Sampling

Sterile cotton swabs were used for sampling the product surface in accordance with ISO 18593:2004 [31]. The swab head was moistened with sterile saline solution, and excess saline was removed by pressing the swab head against the interior wall of a vial with a rotating motion. A sampling template with a 5 cm × 5 cm opening was used to define the sample size and to determine the number of colony forming units (CFU) per cm^2^. After the area was swabbed, the swab head was placed in a vial and a series of dilutions of the sample was made [32]. Only plates (or replicate plates from the same dilution) with 30–300 colonies were counted.

#### 3.4.1. Total Number of Microorganisms of Meat Sample Covered by Hydrosol

The TNM values were determined by examining the colonies formed on agar after incubating the plates at 30 °C for 72 h according to ISO 2293:1988 [33]. The culture medium used in the agar (Merck, Darmstadt, Germany) plate was prepared according to standard procedures using tryptone (BTL Ltd., Lodz, Poland), yeast extract (Merck), and glucose broth (BTL Ltd.). The samples were plated in duplicate and the results were averaged. Colonies grown on the plate were counted automatically after the incubation. The results were expressed as log cfu/cm^2^.

#### 3.4.2. Psychrotrophs

Psychrotrophs were determined on PCA plates (hydrolysed casein (BTL Ltd.), yeast extract, glucose, agar) incubated at 6 °C for 10 days [34]. The samples were plated in duplicate and the results were averaged. The grown colonies were counted automatically at the end of the incubation period. The results were expressed as log cfu/cm^2^.

#### 3.4.3. Number of Yeast and Mold

Culture medium supplemented with chloramphenicol (Sigma-Aldrich, Poznan, Poland) was used for the determination of NYM. Other components of the medium were yeast extract, glucose, and agar. The plates were incubated for 5 days at 25 °C [35] and colonies were counted automatically. Samples were plated in duplicate and the results were averaged and expressed as log cfu/cm^2^.

### 3.5. Colour Measurement

Before and after coating, the meat colour was measured using a Minolta Cr 400 colourimeter. The chroma meter was set at D65 illuminant and 10° standard observer. The results were expressed in the CIE LAB (Commission Internationale de l’Eclairage L* a* b* parameters) colour scale, where L* indicated lightness, a* redness, and b* yellowness. The L* value ranges from 0–100, where 0 represents black and 100 represents a perfect reflecting diffuser. The a* and b* indicate colour directions, where +a* is red; −a* is green; b* is yellow; and −b* is blue.

### 3.6. Thiobarbituric Acid Reactive Substances (TBARS) Measurement

From each treatment group, 10 g of ground pork was homogenized with 25 mL of trichloroacetic acid (TCA) solution for 30 s and filtered. The TCA solution consisted of 200 g/L of TCA dissolved in a 135 mL/L solution of phosphoric acid. Five millilitres of the filtrate was mixed with 2 mL of 20 mM thiobarbituric acid, heated in boiling water for 20 min, cooled in cold water for 10 min, and centrifuged for 15 min at 5500 g. The absorbance at 532 nm was measured using a UV 1800 model spectrophotometer (Rayleigh Instruments Limited, Sevenoaks, UK). The level of TBARS was quantified from the absorbance of the samples with the help of an equation and a standard curve constructed using malondialdehyde (MDA) as a standard. The TBARS values were expressed as mg MDA per kg meat.

### 3.7. Statistical Analysis

This study analysed nine different muscles, 12 experimental meat coating conditions, and the meat slices themselves as controls (*n* = 108) for three analytical repetitions. The experimental design was a randomized design and measurements were made in triplicate. From each muscle, 12 slices of each variant were obtained. The effect of cellulase concentration (0%, 0.05%, 0.1%) and storage time (0, 7, 14 days) on the TNM, P, NYM, colour changes, and lipid oxidation changes of the meat were examined using a two way ANOVA (Statistic 9, StatSoft Poland, Cracow, Poland), where the measured variables were set as dependent variables, cellulase concentration and storage time as fixed effects, and replicates as random effects. The mean values and the standard errors of the mean were reported. The differences between means were established by the Duncan Test with a confidence level set at *p* < 0.05.

## 4. Conclusions

Cellulase was added to the edible coating material consisting of HPMC and CH to improve the quality of stored pork and extend its shelf-life. Cellulase improved the antimicrobial activity of chitosan on the surface of meat. The addition of cellulase led to a significant reduction in the total number of microorganisms and psychotropic bacteria, as well as yeast and mold. The coatings stabilized the a_w_ and pH of the pork meat in cold storage for 14 days. The reduction in TBARS values as well as the slight changes in the a* parameter confirmed the positive effect of cellulase in the coating formation, leading to the production of bioactive polysaccharides oligomers. Meat coated with a mixture of HPMC, CH, and 0.05% cellulase can be stored in cold conditions for 7 days. Doubling the enzyme concentration extends this time to 14 days or longer. Thus, such coatings guarantee meat safety and extend the shelf-life of pork meat.

## Figures and Tables

**Table 1:** Effect of cellulase addition and storage time on the physico-chemical parameters of coated meat.

Interaction Effects C × T * (% × Days)	Parameters					
	pH	a_w_	L*	a*	b*	TBARS ^#^ (mg MDA/kg)
**C0T0**	5.47 ± 0.01 ^ab^	0.925 ± 0.001 ^b^	53.91 ± 0.34 ^ac^	6.89 ± 0.62 ^abc^	4.82 ± 0.20 ^ab^	0.26 ± 0.05 ^a^
**C0.05T0**	5.42 ± 0.02 ^e^	0.926 ± 0.001 ^b^	52.78 ± 0.40 ^a^	5.76 ± 0.65 ^ab^	3.71 ± 0.55 ^af^	0.25 ± 0.01 ^a^
**C0.1T0**	5.43 ± 0.01 ^e^	0.925 ± 0.001 ^b^	51.89 ± 1.82 ^a^	6.20 ± 0.09 ^ab^	2.77 ± 1.01 ^f^	0.26 ± 0.01 ^a^
**C0T7**	5.48 ± 0.01 ^d^	0.921 ± 0.001 ^a^	59.45 ± 0.30 ^bd^	5.47 ± 0.33 ^a^	8.23 ± 0.40 ^de^	0.68 ± 0.02 ^d^
**C0.05T7**	5.51 ± 0.01 ^ac^	0.921 ± 0.000 ^a^	55.13 ± 1.43 ^ac^	6.22 ± 0.21 ^ab^	4.86 ± 0.80 ^ab^	0.66 ± 0.01 ^c^
**C0.1T7**	5.54 ± 0.01 ^b^	0.919 ± 0.000 ^d^	52.61 ± 0.56 ^a^	8.28 ± 0.54 ^c^	7.13 ± 0.29 ^cde^	0.53 ± 0.01 ^b^
**C0T14**	5.47 ± 0.01 ^ac^	0.923 ± 0.000 ^c^	60.61 ± 1.04 ^d^	7.37 ± 0.30 ^bc^	8.85 ± 0.17 ^e^	1.28 ± 0.01 ^h^
**C0.05T14**	5.50 ± 0.02 ^c^	0.922 ± 0.000 ^a^	56.90 ± 0.76 ^bc^	6.85 ± 0.62 ^ab^	5.95 ± 0.92 ^bc^	1.08 ± 0.21 ^g^
**C0.1T14**	5.52 ± 0.01 ^abc^	0.921 ± 0.001 ^a^	59.63 ± 2.02 ^bd^	6.30 ± 0.49 ^ab^	6.83 ± 0.63 ^cd^	1.05 ± 0.02 ^f^
**Control (meat) T0**	5.52 ± 0.00 ^ab^	0.926 ± 0.000 ^b^	58.62 ± 0.06 ^bd^	8.57 ± 1.21 ^c^	0.95 ± 0.32 ^g^	0.26 ± 0.01 ^a^
**Control (meat) T7**	5.52 ± 0.01 ^abc^	0.922 ± 0.001 ^ac^	56.31 ± 0.77 ^bc^	5.69 ± 0.11 ^ab^	5.02 ± 0.24 ^ab^	0.97 ± 0.01 ^e^
**Control (meat) T14**	5.54 ± 0.00 ^b^	0.921 ± 0.000 ^a^	38.01 ± 1.14 ^e^	3.68 ± 0.20 ^d^	7.46 ± 0.27 ^cde^	1.54 ± 0.01 ^i^

Results are expressed as the mean ± standard error. Values with different letters (a–g) within the same column differ significantly (*p* < 0.05). * C = cellulase, T = storage time of sample. ^#^ TBARS—Thiobarbituric Acid Reactive Substances.

**Table 2:** Experimental design.

Variants Coding *	Variables		Constant Factors				
	Cellulase (%)	Time (Days)	Chitosan (%)	HPMC (%)	Glycerol (%)	Lactic Acid (%)	Sample
C0T0	0	0	1	1	25	0.25	Meat slice
C0.05T0	0.05	0	1	1	25	0.25	Meat slice
C0.1T0	0.1	0	1	1	25	0.25	Meat slice
C0T7	0	7	1	1	25	0.25	Meat slice
C0.05T7	0.05	7	1	1	25	0.25	Meat slice
C0.1T7	0.1	7	1	1	25	0.25	Meat slice
C0T14	0	14	1	1	25	0.25	Meat slice
C0.05T14	0.05	14	1	1	25	0.25	Meat slice
C0.1T14	0.1	14	1	1	25	0.25	Meat slice
Control (meat) T0	-	0	-	-	-	-	Meat slice
Control (meat) T7	-	7	-	-	-	-	Meat slice
Control (meat) T14	-	14	-	-	-	-	Meat slice

- Not applied. * C = cellulase, T = storage time of sample.
